# Evaluation of angle-to-angle and spur-to-spur using swept source optical coherence tomography in different refractive error

**DOI:** 10.1371/journal.pone.0277703

**Published:** 2022-11-21

**Authors:** Jeong Seop Yun, Ji Sang Min, Kook Young Kim

**Affiliations:** 1 Kim’s Eye Hospital, Seoul, Korea; 2 Department of Ophthalmology, Institute of Vision Research, Yonsei University College of Medicine, Seoul, South Korea; 3 Nuri Eye Hospital, Deajeon, Korea; Nicolaus Copernicus University, POLAND

## Abstract

**Purpose:**

To measure angle-to-angle (ATA) and spur-to-spur (STS) distances along six meridians using swept-source optical coherence tomography (SS-OCT) and compare with horizontal white-to-white (WTW) distance in different refractive error.

**Methods:**

Overall, 126 eyes were assessed with the Anterion SS-OCT (Heidelberg Engineering, Heidelberg, Germany). ATA and STS distances were obtained using SS-OCT at 0, 30, 60, 90, 120, and 150 degrees. WTW was measured at 0 degree with built-in infrared camera. One way ANOVA test, pearson correlation coefficient, and stepwise multivariate regression analysis were used to compare ATA and STS distances with age, anterior chamber depth (ACD), axial length (AL), and simulated keratometric values (Sim K) in different refractive error groups.

**Results:**

The mean MRSE refraction was +0.05 ± 0.23 D in the emmetropic group (41 eyes), -3.42 ± 3.04 D in the myopic group (44 eyes), and +1.33 ± 0.64 D in the hyperopic group (31 eyes). There was no statistical difference in the WTW of the emmetropic (11.62 ± 0.44 mm), myopic (11.79 ± 0.46 mm), and hyperopic groups (11.80 ± 0.49 mm) using one-way ANOVA (p = 0.007). ATA and STS were vertically oval in all groups. The correlation between ATA, STS and age, ACD, AL, and K values showed different significance for each meridian according to the refractive error. ATA increased as the horizontal WTW, ACD, and AL increased and Sim K decreased. STS shows relatively smaller explanatory power than ATA in the stepwise multivariate regression analysis.

**Conclusions:**

This study is the first to analyze the relationship between ATA and STS compared to WTW by different refractive error. The difference between the horizontally oval WTW and vertically oval anterior chamber can be large, especially in myopia. ATA showed a greater positive correlation than STS with AL and ACD.

## Introduction

The development of biometric measurement makes it possible to accurately measure the anterior segment structure. The anterior chamber structure including the ciliary sulcus distance, anterior chamber depth (ACD), and angle to angle distance (ATA) are important measures for phakic intraocular lens implantation [1–3]. In addition, the corneal limbus is used to estimate the port position during vitrectomy or suture position during intraocular lens scleral fixation. Furthermore, it is used as a reference point for locating the trabecular meshwork (TM) during glaucoma laser treatment such as selective laser trabeculoplasty.

The identification of the position of the anterior angle or the size of the ciliary sulcus was classically assessed through measurement of white to white (WTW), which is the horizontal distance between the borders of the corneal limbus. However, the WTW may not always accurately represent the angle, sulcus position, and TM.

Recently, high-resolution swept-source optical coherence tomography (SS-OCT) has been introduced to analyze not only the posterior segments but also the anterior segment of the eyeball [4, 5]. It can analyze the corneal topography, the axial length (AL), and actual assessment of angle structures including the angle recess and scleral spur owing to the greater tissue penetration depth by the light source [6, 7].

In a previous study, WTW predicted the sulcus-to-sulcus diameter, and there was a difference between myopia and emmetropia [8]. Prediction of sulcus-to-sulcus diameter with WTW in highly myopic eyes showed different correlations according to ACD [9].

This study aims to evaluate the relationship between the horizontal WTW distance, and the actual angle location, represented by angle-to-angle distance (ATA) and spur to spur distance (STS) between different refractive errors using new type SS-OCT.

## Material and methods

We retrospectively reviewed the medical records of patients who had undergone SS-OCT at Kim’s Eye Hospital from June 2020 to July 2021. The study protocol was approved by the Institutional Review Board (IRB number: 2021-12-004) at Kim’s Eye Hospital, Seoul, Korea, and the study was conducted in accordance with the tenets of the Declaration of Helsinki.

This study included 126 eyes, and only one eye from each particapant was randomly selected for the study. The exclusion criteria were as follows: ophthalmologic diseases that could affect the measurement of anterior segment, such as poor fixation; a history of ocular surgery, corneal disease, glaucoma, or retinal disease; dense cataracts that could affect refractive error; and cataracts with a corrected distance visual acuity (CDVA) of less than 0.7 (decimal value). All participants’ eyes were phakic. CDVA was measured with decimal values on the Snellen visual acuity chart. All eyes were examined using manifest refraction and classified into three refractive status groups according to the manifest refraction spherical equivalent (MRSE) refractive error: myopia (≦ −0.5 D), emmetropia (> −0.5 to < 0.5 D), and hyperopia (≧ +0.5 D).

**Fig 1:**
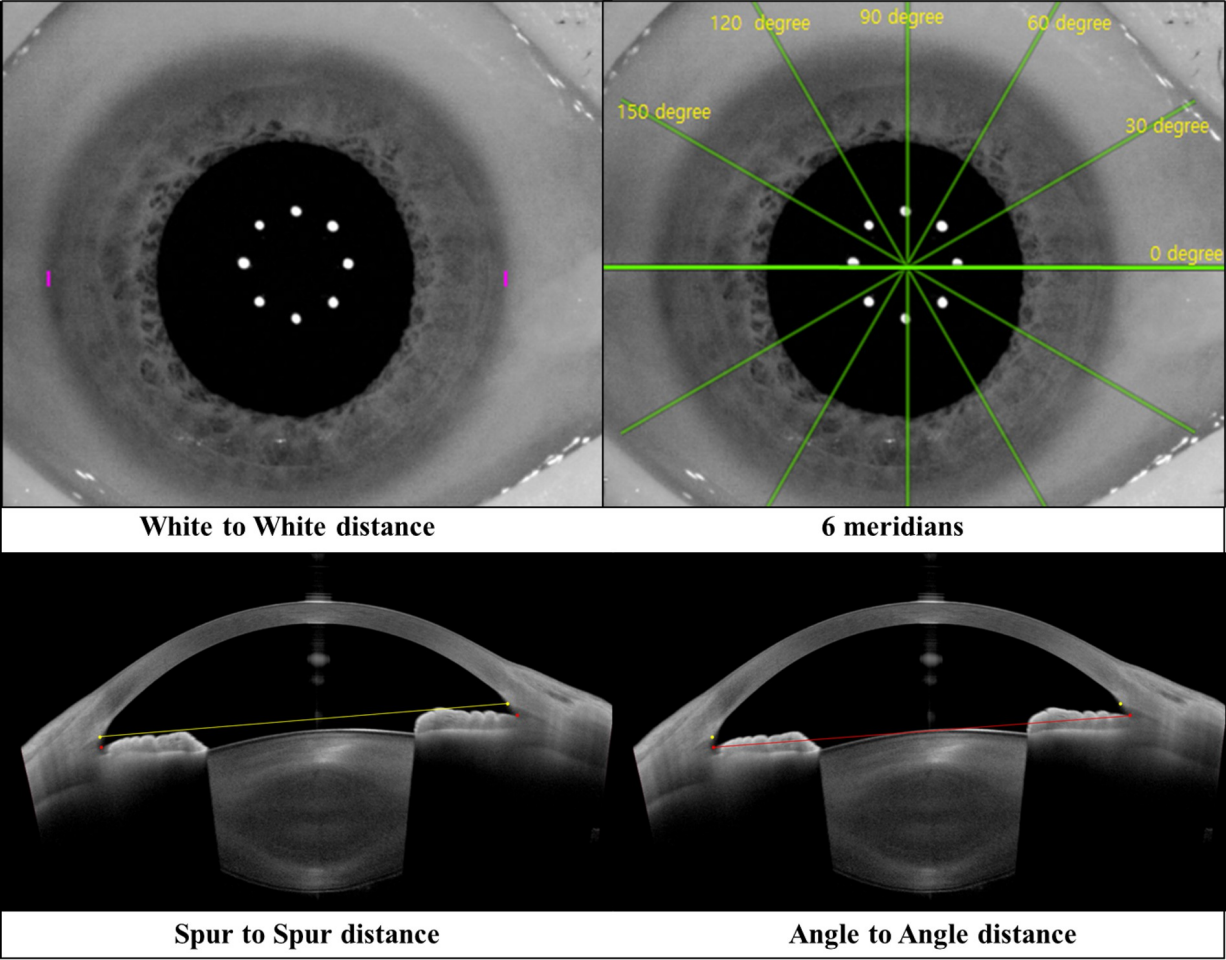
White to white, spur to spur, and angle to angle distances at the 0 degree (horizontal) meridian, and 6 meridians measured with the anterion swept source optical coherence tomography.

The ANTERION (Heidelberg Engineering Inc., Heidelberg, Germany) is a new high-resolution SS-OCT device capable of capturing a wider scan depth (14.5 mm) and scan width (16.5 mm), with a light source of 1300-nm wavelength, compared to the existing SS-OCT devices, and of measuring the AL in the range of 14–32 mm. The use of a long wavelength makes it possible to image the whole anterior segment including lens and the lateral scanning SS-OCT allows for cross-sectional imaging providing data of different parameters analyzed. The following anterior segment parameters were measured by CATARACT mode and METRIC mode using a lateral scanning SS-OCT and an infrared camera (820 to 890 nm LED light source): white-to-white distance (WTW) was defined as the horizontal distance between the nasal and temporal limbus, measured by infrared camera image, angle-to-angle distance (ATA) was defined as the distance from angle recess point to angle recess point in one cross-sectional B-scan, spur-to spur distance (STS) was defined as the distance between one scleral spur to the other scleral spur within one cross-sectional B-scan. Measurements were taken following the manufacturer’s instructions. Before the test, the patients were instructed to fix their heads accurately and blink their eyes so that tears were uniformly applied to the corneal surface. Each patient was correctly positioned on the chin rest with the forehead reclining on the ANTERION SS-OCT. The test results confirmed that the three acquisition quality parameters (“Motion,” “Fixation,” and “Tear film and lid”) all received a "pass" score. If any measurements did not receive a “pass,” the examination was repeated. WTW was measured in only the horizontal meridian, and ATA and STS were measured along 6 meridians: 0 degree (3–9 o’clock, horizontal), 30 degree (2–8 o’clock), 60 degree (1–7 o’clock), 90 degree (6–12 o’clock, vertical), 120 degree (5–11 o’clock), and 150 degree (4–10 o’clock) (Fig 1).

### Statistical analysis

Data were analyzed using SPSS version 24.0 (IBM Corporation, Armonk, NY, USA). The Shapiro–Wilk test was used to evaluate the normality of numerical data. The one-way analysis of variance (ANOVA) was used if variables were normally distributed, whereas the Kruskal–Wallis test was used if one or more variables were not normally distributed. Bonferroni’s post-hoc test was used to compare the WTW, STS and ATA in three different refractive error groups. Pearson’s correlation coefficient was used to analyze the correlation between ATA and STS with age, AL, ACD, and Sim K at different meridians by refractive error. Stepwise multivariate regression analysis was constructed with ATA and STS as the dependent variables and age, MRSE, axial length, ACD, and Sim K as the covariates. Statistical significance was set at p < 0.05.

## Results

This study included 126 eyes of 126 participants. The mean age was 53.04 ± 14.65 years (range from 19–83 years). Approximately 44 eyes were myopic, 41 eyes were emmetropic, and 41 eyes were hyperopic. Except for Sim K (steep and flat), most of the clinical characteristics showed significant differences (p < 0.05) according to refractive error (Table 1).

**Table 1:** Clinical characteristics.

	Total (N = 126)	Myopia (N = 44)	Emmetropia (N = 41)	Hyperopia (N = 41)	
	Mean ± SD	Mean ± SD	Mean ± SD	Mean ± SD	P-value
**Age, year**	53.04 ± 14.65	45.30 ± 15.83	53.83 ± 10.99	60.56 ± 12.46	0.000**
**MRSE, D**	-0.75 ± 2.73	-3.42 ± 3.04	0.05 ± 0.23	1.33 ± 0.64	0.000**
**CDVA**	0.93 ± 0.10	0.92 ± 0.13	0.95 ± 0.08	0.93 ± 0.10	0.328**
**AL, mm**	23.77 ± 1.16	24.66 ± 1.10	23.47 ± 0.63	23.09 ± 1.05	0.000*
**Sim K-steep, D**	43.98 ± 1.67	44.41 ± 1.88	43.78 ± 1.27	43.98 ± 1.67	0.105*
**Sim K-flat, D**	43.17 ± 1.41	43.39 ± 1.37	43.07 ± 1.21	43.04 ± 1.64	0.465*
**ACD, mm**	3.27 ± 0.42	2.96 ± 0.40	2.70 ± 0.43	2.50 ± 0.27	0.000*
**Lens thickness, mm**	4.45 ± 0.47	4.17 ± 0.46	4.55 ± 0.40	4.71 ± 0.35	0.000*

* One-way ANOVA test. P < 0.05 is statistically significant.

** Kruskal Wallis test. P < 0.05 is statistically significant.

CDVA, Corrected distance visual acuity; MRSE, manifest refraction spherical equivalent; AL, axial length; Sim K, simulated keratometry; ACD, anterior chamber depth

There was statistical difference in WTW by refractive error (One-way ANOVA, p = 0.007) (Table 2). In Bonferroni’s post hoc test, there were no significant differences between the myopia and hyperopia groups (p = 1.000); all other post hoc tests showed significant differences. The horizontal WTW was positively correlated with AL (r = 0.254) and ACD (r = 0.264) and negatively correlated with age (r = -0.151) and Sim K (steep r = -0.257 and flat r = -0.330) (all p < 0.05) in 126 eyes. In the stepwise multivariate regression analysis on WTW with 6 variables (age, MRSE, ACD, Sim K steep, Sim K flat, and AL), a model consisting of 2 variables (standardized partial regression coefficient β: ACD: 0.267 and Sim K flat: -0.332; p < 0.001) explained only 16.7% of the WTW variability. In ATA, there were statistical differences in all meridians by refractive error (One-way ANOVA, p < 0.05). In the Bonferroni’s post-hoc test, ATA of the myopia-emmetropia and emmetropia-hyperopia group showed significant differences except for ATA 120 (p > 0.055) in all meridians; however, there were no significant differences in all other meridians in the analysis of the emmetropia-hyperopia groups. Unlike ATA, which showed a significant difference in all three groups, there was no significant difference in STS 120 (p = 0.051), and there was a significant difference between the three groups in the other meridians. The post hoc analysis showed statistical significance similar to that of ATA in myopia-emmetropia group; however, there was no significant difference in the myopia-hyperopia and emmetropia-hypertropia group in all meridians.

**Table 2:** WTW, ATA, and STS distances at different meridians.

	Total (N = 126)	Myopia (N = 44)	Emmetropia (N = 41)	Hyperopia (N = 41)				
	Mean ± SD (range), mm	Mean ± SD (range), mm	Mean ± SD (range), mm	Mean ± SD (range), mm	P^+^	P^a^	P^b^	P^c^
**WTW**	11.71 ± 0.47 (10.66 to 13.04)	11.79 ± 0.46 (10.66 to 12.81)	11.62 ± 0.44 (10.82 to 12.72)	11.80 ± 0.49 (10.84 to 13.04)	0.007	0.020	1.000	0.017
**ATA-0**	11.69 ± 0.44 (10.72 to 12.89)	11.86 ± 0.41 (10.94 to 12.62)	11.58 ± 0.44 (10.72 to 12.48)	11.62 ± 0.42 (10.74 to 12.89)	0.005	0.008	0.031	1.000
**STS-0**	11.52 ± 0.39 (10.50 to 12.63)	11.65 ± 0.37 (10.84 to 12.50)	11.41 ± 0.36 (10.68 to 12.02)	11.48 ± 0.40 (10.50 to 12.63)	0.015	0.016	0.125	1.000
**ATA-30**	11.59 ± 0.40 (10.68 to 12.49)	11.73 ± 0.39 (10.81 to 12.49)	11.48 ± 0.39 (10.68 to 12.33)	11.55 ± 0.39 (10.75 to 12.48)	0.014	0.014	0.123	1.000
**STS-30**	11.46 ± 0.37 (10.51 to 12.33)	11.58 ± 0.35 (10.62 to 12.27)	11.34 ± 0.32 (10.70 to 12.23)	11.43 ± 0.40 (10.51 to 12.33)	0.013	0.010	0.231	0.718
**ATA-60**	11.79 ± 0.44 (10.25 to 13.09)	11.95 ± 0.39 (11.02 to 12.92)	11.69 ± 0.44 (10.25 to 12.55)	11.72 ± 0.46 (10.90 to 13.09)	0.012	0.022	0.046	1.000
**STS-60**	11.66 ± 0.39 (10.70 to 12.60)	11.81 ± 0.36 (10.70 to 12.48)	11.54 ± 0.34 (10.82 to 12.35)	11.63 ± 0.43 (10.81 to 12.61)	0.004	0.003	0.098	0.748
**ATA-90**	11.95 ± 0.53 (10.75 to 13.24)	12.14 ± 0.49 (10.91 to 12.94)	11.85 ± 0.50 (10.80 to 12.92)	11.85 ± 0.56 (10.75 to 13.24)	0.014	0.036	0.035	1.000
**STS-90**	11.79 ± 0.47 (10.54 to 13.11)	11.96 ± 0.42 (10.54 to 12.71)	11.67 ± 0.38 (10.78 to 12.44)	11.73 ± 0.55 (10.68 to 13.11)	0.009	0.010	0.079	1.000
**ATA-120**	11.82 ± 0.47 (10.83 to 13.26)	11.98 ± 0.43 (11.02 to 12.80)	11.75 ± 0.42 (10.93 to 12.74)	11.73 ± 0.52 (10.83 to 13.26)	0.020	0.055	0.040	1.000
**STS-120**	11.65 ± 0.44 (10.53 to 13.03)	11.78 ± 0.43 (10.53 to 12.52)	11.58 ± 0.34 (10.89 to 12.34)	11.57 ± 0.52 (10.62 to 13.03)	0.051	0.106	0.103	1.000
**ATA-150**	11.62 ± 0.43 (10.45 to 12.68)	11.76 ± 0.40 (10.87 to 12.64)	11.52 ± 0.42 (10.65 to 12.34)	11.56 ± 0.42 (10.45 to 12.68)	0.018	0.027	0.079	1.000
**STS-150**	11.50 ± 0.39 (10.43 to 12.75)	11.62 ± 0.41 (10.64 to 12.75)	11.39 ± 0.33 (10.69 to 12.25)	11.49 ± 0.41 (10.43 to 12.65)	0.035	0.031	0.383	0.879

WTW, white-to-white distance; ATA, angle-to-angle distance; STS, spur-to-spur distance.

* One-way ANOVA test. *P* < 0.05 is statistically significant.

a Bonferroni post hoc test between myopia and emmetropia. *P* < 0.05 is statistically significant.

b Bonferroni post hoc test between myopia and hyperopia. *P* < 0.05 is statistically significant.

c Bonferroni post hoc test between emmetropia and hyperopia. *P* < 0.05 is statistically significant.

**Fig 2:**
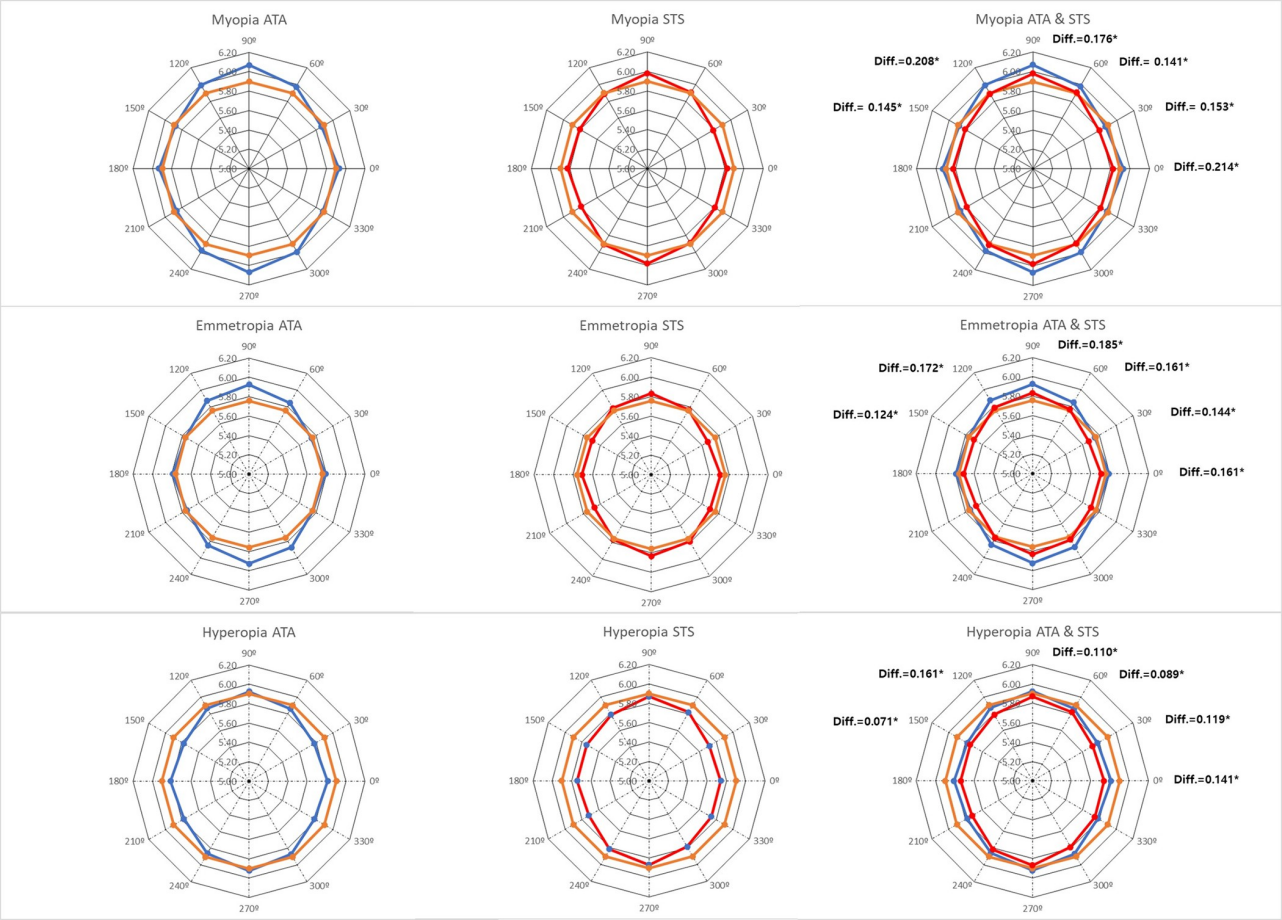
Angle to angle (blue lines) and spur to spur (red line) distances measured at the different meridians. Horizontal white to white was added and shown as orange lines for comparison. Radial axis starts at 5 mm. Diff. means the difference in value between the ATA and the STS.

In a radial graphical representation for each meridian at different refractive error, compared to the WTW measured only horizontal meridian (expressed as orange lines on Fig 2), ATA and STS distances show that the anterior chamber is vertically oval regardless of refractive error (Fig 2). In the myopia group and emmetropia group, ATA and STS on the vertical meridian were larger and ATA and STS on the horizontal meridian were smaller than the horizontal WTW in each group. In the hyperopia group, only the vertical ATA 90 was larger, and the STS 90 was slightly smaller than the horizontal WTW. The horizontal ATA and STS were both smaller than the WTW. Although the vertical oval pattern of ATA and STS was the same in both the myopia and emmetropia groups, the size distribution compared to WTW was different. In all groups, ATA was larger than STS in all meridians, showing a significant difference.

Significant correlations were shown between WTW, ATA, and STS in all meridians in the myopia, emmetropia, and hyperopia groups; however, different correlation values were shown for each group and each meridian (Table 3). Overall, it showed the lower the correlation with WTW in the closer to the vertical axis, and the higher the correlation in the closer to the horizontal axis. In the hyperopia group, the correlation on the horizontal axis was larger than in the other refractive groups.

**Table 3:** Pearson correlation coefficient for horizontal white to white (WTW) versus angle to angle (ATA) and spur to spur (STS) measured at different meridians in each refractive group. All results had a statistical significance (p < 0.05).

**ATA**	**WTW-ATA0**	**WTW-ATA30**	**WTW-ATA60**	**WTW-ATA90**	**WTW-ATA120**	**WTW-ATA150**
**Myopia**	0.408	0.320	0.285	0.248	0.242	0.380
**Emmetropia**	0.248	0.301	0.128	0.200	0.216	0.284
**Hyperopia**	0.473	0.434	0.164	0.230	0.221	0.435
**STS**	**WTW-STS0**	**WTW- STS30**	**WTW- STS60**	**WTW- STS90**	**WTW- STS120**	**WTW- STS150**
**Myopia**	0.392	0.337	0.278	0.271	0.255	0.331
**Emmetropia**	0.249	0.234	0.155	0.158	0.092	0.218
**Hyperopia**	0.526	0.493	0.135	0.175	0.165	0.406

**Fig 3:**
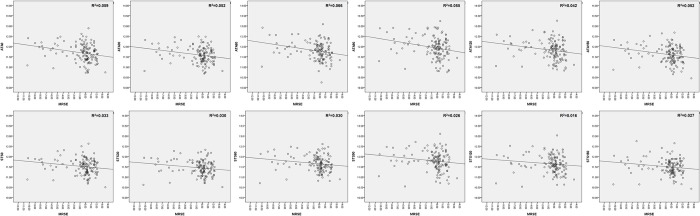
Scatter plots showing the relationship between angle to angle (ATA) and spur to spur (STS) versus manifest refractive spherical error (MRSE) in study population (126 eyes).

Fig 3 is a scatter plot of ATA and STS for each MRSE along each meridian in total 126 eyes. Although there is a difference for each meridian, the R^2^ value did not exceed 0.1 in all meridian.

Table 4 shows the correlation between WTW and ATA values for each refractive group according to age, AL, ACD, and corneal curvature. All groups showed an overall negative correlation with age; only the emmetropia group showed a significant negative correlation. The AL and ACD showed a significant positive correlation with ATA in all groups. In the corneal curvature, there was no significant correlation in the myopia group, and there was a significant negative correlation in some meridians (ATA-60 and ATA-90 in Sim K steep, and ATA-0 and ATA-90 in Sim K flat) in the emetropic group. In the hyperopia group, there was a significant negative correlation in all meridians.

**Table 4:** Correlation between ATA with age, axial length, anterior chamber depth, and Sim K at different meridians by refractive error.

	ATA0		ATA30		ATA60		ATA90		ATA120		ATA150	
	R	P*	R	P*	R	P*	R	P*	R	P*	R	P*
**Age**												
Myopia	-0.200	0.096	-0.136	0.190	-0.239	0.059	-0.243	0.056	-0.180	0.121	-0.130	0.200
Emmetropia	-0.321	0.020	-0.310	0.024	-0.294	0.031	-0.373	0.008	-0.350	0.012	-0.362	0.010
Hyperopia	-0.081	0.307	0.053	0.371	-0.043	0.395	-0.108	0.251	-0.162	0.156	-0.081	0.307
**Axial length**												
Myopia	0.360	0.008	0.466	0.001	0.438	0.001	0.397	0.004	0.261	0.043	0.394	0.004
Emmetropia	0.383	0.007	0.408	0.004	0.475	0.001	0.592	<0.001	0.460	0.001	0.413	0.004
Hyperopia	0.589	<0.001	0.632	<0.001	0.516	0.001	0.453	0.001	0.460	0.001	0.611	<0.001
**ACD**												
Myopia	0.425	0.002	0.451	0.001	0.526	<0.001	0.538	<0.001	0.430	0.002	0.468	0.001
Emmetropia	0.584	<0.001	0.653	<0.001	0.664	<0.001	0.675	<0.001	0.663	<0.001	0.678	<0.001
Hyperopia	0.493	0.001	0.515	0.001	0.436	0.002	0.462	0.001	0.436	0.002	0.504	0.001
**Sim K steep**												
Myopia	-0.114	0.231	-0.149	0.168	-0.094	0.273	-0.166	0.140	-0.223	0.073	-0.146	0.172
Emmetropia	-0.216	0.087	-0.257	0.053	-0.262	0.049	-0.374	0.008	-0.198	0.107	-0.214	0.089
Hyperopia	-0.519	0.001	-0.593	<0.001	-0.546	<0.001	-0.451	0.002	-0.477	0.001	-0.599	<0.001
**Sim K flat**												
Myopia	-0.241	0.058	-0.216	0.080	-0.052	0.368	-0.093	0.274	-0.120	0.219	-0.167	0.139
Emmetropia	-0.274	0.042	-0.242	0.063	-0.231	0.073	-0.336	0.016	-0.215	0.089	-0.249	0.058
Hyperopia	-0.579	<0.001	-0.660	<0.001	-0.561	<0.001	-0.448	0.002	-0.455	0.001	-0.610	<0.001

WTW, white-to-white distance; ATA, angle-to-angle distance; STS, spur-to-spur distance; ACD, anterior chamber depth; sim K, simulated keratometry.

* Pearson correlation. *P* < 0.05 is statistically significant.

Except for STS-0 in the emmetropic group, there was no significant correlation with age in any other meridians. In the AL, a significant positive correlation was shown in all meridians in the hyperopia group, but only in some meridians (STS-0 and STS-30) in the myopic group and emmetropic group. ACD showed a significant positive correlation with STS in all groups except for STS-90 and STS-120 in the hyperopia group. The correlation between corneal curvature and STS showed a significant negative correlation in the hyperopia group, and a significant negative correlation only in some meridians in the myopia and emmetropia group (Table 5). The correlation between WTW and age (R^2^ = -0.151), AL (R^2^ = 0.254), MRSE, ACD (R^2^ = 0.264), and corneal curvature (Sim K steep R^2^ = -0.257 and Sim K flat R^2^ = -0.330) was significant except for MRSE. In stepwise multiple regression analysis with the above factors, only Sim K flat and ACD appeared as significant variables, and their explanatory power was 16.7%. (WTW = 15.553–0.112 * Sim K flat +0.301 * ACD, adjusted R^2^ = 0.167, p<0.001). The results of the stepwise multivariate linear regression analysis for ATA and STS of each meridian as the dependent variable with age, axial length, MRSE, WTW, ACD, and Sim K (steep and flat) are summarized in Tables 6 and 7. ATA showed explanatory power composed of different variables for each meridian, but both WTW and ACD showed high coefficient values, and the closer to the horizontal meridian, the greater the explanatory power compared to the vertical meridian. ATA increased as the horizontal WTW, ACD, and AL increased, and Sim K decreased. STS shows relatively smaller explanatory power at each meridian than ATA, and as in ATA analysis, WTW shows the highest coefficient value but tends to be composed of variables different from ATA.

**Table 5:** Correlation between STS with age, axial length, anterior chamber depth, and Sim K at different meridians by refractive error.

	STS0		STS30		STS60		STS90		STS120		STS150	
	R	P*	R	P*	R	P*	R	P*	R	P*	R	P*
**Age**												
Myopia	-0.128	0.203	-0.069	0.329	-0.160	0.149	-0.139	0.183	-0.108	0.243	-0.045	0.386
Emmetropia	-0.295	0.030	-0.190	0.117	-0.079	0.312	-0.134	0.202	-0.246	0.061	-0.211	0.092
Hyperopia	0.044	0.392	0.084	0.300	0.184	0.124	0.083	0.304	0.028	0.431	0.066	0.341
**Axial length**												
Myopia	0.301	0.038	0.315	0.019	0.187	0.111	0.093	0.275	0.082	0.301	0.204	0.092
Emmetropia	0.329	0.018	0.229	0.075	0.067	0.338	0.196	0.110	0.002	0.495	0.020	0.445
Hyperopia	0.578	<0.001	0.656	<0.001	0.585	<0.001	0.492	0.001	0.433	0.002	0.639	<0.001
**ACD**												
Myopia	0.271	0.038	0.308	0.021	0.350	0.010	0.321	0.017	0.251	0.050	0.327	0.015
Emmetropia	0.511	0.001	0.407	0.004	0.269	0.045	0.294	0.031	0.348	0.013	0.370	0.009
Hyperopia	0.423	0.003	0.399	0.005	0.285	0.036	0.196	0.110	0.214	0.089	0.357	0.011
**Sim K steep**												
Myopia	-0.167	0.139	-0.220	0.076	-0.257	0.046	-0.265	0.041	-0.216	0.079	-0.174	0.130
Emmetropia	-0.215	0.089	-0.207	0.098	-0.114	0.238	-0.195	0.111	0.070	0.331	0.019	0.453
Hyperopia	-0.576	<0.001	-0.533	<0.001	-0.632	<0.001	-0.560	<0.001	-0.556	<0.001	-0.670	<0.001
**Sim K flat**												
Myopia	-0.297	0.025	-0.291	0.028	-0.197	0.099	-0.166	0.141	-0.165	0.143	-0.199	0.098
Emmetropia	-0.296	0.020	-0.183	0.125	-0.037	0.409	-0.136	0.199	0.039	0.403	-0.036	0.411
Hyperopia	-0.582	<0.001	-0.594	<0.001	-0.588	<0.001	-0.489	<0.001	-0.485	0.001	-0.637	<0.001

WTW, white-to-white distance; ATA, angle-to-angle distance; STS, spur-to-spur distance; ACD, anterior chamber depth; sim K, simulated keratometry.

* Pearson correlation. *P* < 0.05 is statistically significant.

**Table 6:** Results of the stepwise multivariate regression analysis with age, axial length, manifest refraction spherical equivalent, horizontal WTW, anterior chamber depth, and Sim K (steep and flat) at angle-to-angle diameter.

N = 126	Parameters	Partial Regression Coefficient (B)	Standardized Partial Regression Coefficient (β)	p-value
**ATA 0**		(R = 0.754, R^2^ = 0.568, Adjusted R^2^ = 0.554)		
	WTW	0.398	0.430	<0.001
	ACD	0.345	0.331	<0.001
	Sim K_flat	-0.048	-0.153	0.021
	AL	0.060	0.159	0.041
	Constant	6.539		<0.001
**ATA 30**		(R = 0.770, R^2^ = 0.592, Adjusted R^2^ = 0.579)		
	WTW	0.340	0.404	<0.001
	ACD	0.326	0.345	<0.001
	Sim K_flat	-0.050	-0.177	0.006
	AL	0.063	0.183	0.016
	Constant	7.225		<0.001
**ATA 60**		(R = 0.698, R^2^ = 0.487, Adjusted R^2^ = 0.470)		
	WTW	0.209	0.224	0.002
	ACD	0.439	0.418	<0.001
	Sim K_steep	-0.055	-0.208	0.003
	AL	0.075	0.197	0.017
	Constant	8.554		<0.001
**ATA 90**		(R = 0.722, R^2^ = 0.522, Adjusted R^2^ = 0.506)		
	WTW	0.291	0.261	<0.001
	ACD	0.554	0.440	<0.001
	Sim K_steep	-0.070	-0.221	0.001
	AL	0.076	0.167	0.036
	Constant	7.999		<0.001
**ATA 120**		(R = 0.669, R^2^ = 0.448, Adjusted R^2^ = 0.434)		
	WTW	0.286	0.291	<0.001
	ACD	0.531	0.478	<0.001
	Sim K_steep	-0.061	-0.177	0.002
	Constant	9.432		<0.001
**ATA 150**		(R = 0.775, R^2^ = 0.600, Adjusted R^2^ = 0.587)		
	WTW	0.370	0.415	<0.001
	ACD	0.384	0.383	<0.001
	Sim K_steep	-0.045	-0.178	0.004
	AL	0.061	0.166	0.023
	Constant	6.571		<0.001

**Table 7:** Results of the stepwise multivariate regression analysis with age, axial length, manifest refraction spherical equivalent, horizontal WTW, anterior chamber depth, and Sim K (steep and flat) at spur-to spur diameter.

N = 126	Parameters	Partial Regression Coefficient (B)	Standardized Partial Regression Coefficient (β)	p-value
**STS 0**		(R = 0.725, R^2^ = 0.526, Adjusted R^2^ = 0.511)		
	WTW	0.384	0.473	<0.001
	ACD	0.186	0.204	0.012
	Sim K_flat	-0.048	-0.175	0.012
	AL	0.057	0.172	0.034
	Constant	7.124		<0.001
**STS 30**		(R = 0.704, R^2^ = 0.495, Adjusted R^2^ = 0.483)		
	WTW	0.385	0.493	<0.001
	Sim K_steep	-0.032	-0.142	0.035
	AL	0.106	0.331	<0.001
	Constant	5.822		<0.001
**STS 60**		(R = 0.571, R^2^ = 0.326, Adjusted R^2^ = 0.309)		
	WTW	0.267	0.324	<0.001
	Sim K_steep	-0.047	-0.200	0.011
	AL	0.103	0.305	<0.001
	Constant	8.147		<0.001
**STS 90**		(R = 0.584, R^2^ = 0.341, Adjusted R^2^ = 0.319)		
	WTW	0.360	0.365	<0.001
	Sim K_steep	-0.138	-0.492	0.001
	Sim K_flat	0.116	0.348	0.027
	AL	0.132	0.327	<0.001
	Constant	5.525		<0.001
**STS 120**		(R = 0.496, R^2^ = 0.246, Adjusted R^2^ = 0.228)		
	WTW	0.281	0.302	0.001
	ACD	0.258	0.246	0.004
	Sim K_steep	-0.049	-0.187	0.025
	Constant	9.687		<0.001
**STS 150**		(R = 0.626, R^2^ = 0.392, Adjusted R^2^ = 0.382)		
	WTW	0.430	0.520	<0.001
	AL	0.082	0.241	0.001
	Constant	4.535		<0.001

## Discussion

This study revealed that both ATA and STS showed a vertically oval pattern compared to the horizontal WTW but had a different distribution according to the refractive errors. The results demonstrated a significant correlation between WTW and the STS and ATA distances but showed different correlations by refractive error and meridian. In addition, we presented a statistical model for predicting ATA and STS using horizontal WTW, MRSE, ACD, corneal curvature, and AL as variables for each meridian.

In this study, there were significant differences in the mean WTW values for the myopic, emmetropic, and hyperopic groups. However, the mean WTW did not decrease from the myopia group to the hyperopia group, and the mean WTW was greatest in the hyperopic group and the smallest in the emmetropic group. In a previous study using partial coherence interferometry biometer (emmetropia: 12.24 ± 0.57 mm, myopia: 12.17 ± 0.26 mm, p-values = 0.89) and scanning-slit topography 0.37 (emmetropia: 11.83 ± 0.48 mm, myopia: 11.66 ± 0.25 mm, p-values = 0.37), there was no significant difference in WTW between the myopic group and the emmetropic group [8]. In Asian studies with over 30,000 eyes, the mean WTW was 11.69 ± 0.46 mm measured by IOLMaster 700; additionally, there was no linear correlation according to the axial length, and a larger WTW distance was associated with younger age, male sex, larger corneal curvature, ACD, lens thickness, and thinner central corneal thickness [10]. Due to the correlation with these various ocular measurements, a linear correlation with a refractive error similar to that with AL is not expected. In an interracial comparative study conducted by Qin et al. using time-domain OCT, for Asian eyes, WTW was 0.5 mm narrower (P < 0.01), STS was 0.46 mm narrower (P < 0.01), and corneal vault was 0.22 mm lower (P < 0.01) compared to Caucasian eyes [11]. In this study, WTW was found to be a significant explanatory variable for Sim K flat and ACD, but they showed low explanatory power of 16.7% in the stepwise multivariate regression analysis. The reason for this difference is thought to be that WTW is not only affected by various ocular measurements but is also affected by various factors such as differences in measuring instruments, race, age groups, and sex.

ATA and STS did not show similar linear correlations for each refractive group in all meridians. In both ATA and STS of all refractive error groups, the largest values were in the vertical meridian and the shortest were in the 30-degree meridian. In previous studies analyzed with 35 MHz ultrasound biomicroscopy (UBM) [12], 50 MHz UBM [13], and SS-OCT [14], the ATA was greater in the vertical meridian than in the horizontal. Likewise, in the STS, it was greater in the vertical meridian than in the horizontal [14, 15]. Montés-Micó et al. analyzed 68 eyes with ANTERION and showed how ATA and STS change radially, indicating that the vertical meridian is the longest (ATA 12.38 mm, STS 12.33 mm) and the 30-degree meridian is the shortest (ATA 11.88 mm, STS 11.87 mm), both for ATA and STS distances [14]. Bruner et al. analyzed 65 eyes with CASIA SS-1000 and showed that the mean vertical STS was 12.11 ± 0.33 mm, and the mean horizontal STS was 11.87 ± 0.33 mm. [15] Baikoff et al. showed that vertical ATA was greater than horizontal ATA by at least 0.1mm in 74% of the eyes and by more than 0.3 mm in nearly 50% of the eyes [16]. The ellipsoid shape of the anterior chamber, with a larger vertical diameter, is well documented in these previous studies [14–16]. Our results indicated that the anterior chamber is vertically oval in all refractive errors, which is consistent with the results of quite a few previous studies.

A radial graphical representation for each meridian in different refractive errors is shown (Fig 2). However, the size and position patterns were different for each refractive error. In myopia and emmetropia, the ellipse of ATA met the circle of WTW on the horizontal plane, and the ellipse of STS met the circle of WTW at the 60-degree meridian. Contrastingly, in hyperopia, the ellipse of ATA showed a small ellipse that met the circle of WTW at the 120-degree meridian, and STS showed an ellipse that met the circle of WTW at the vertical meridian. Predicting ATA and STS using conventional methods with horizontal WTW is known to be inaccurate. In Table 3, the correlations between WTW and ATA or STS for each meridian are all statistically significant but appear differently for each meridian. Montés-Micó et al. showed the correlation between the horizontal WTW distance and the ATA and STS distances, respectively, obtained along different meridians, (R^2^ ranged from 0.49 to 0.75) [14]. Although the correlation value was larger than in our study, the correlation trend was the largest on the horizontal meridian and decreased toward the vertical meridian, showing a similar pattern. Some previous studies [17–19] concluded that the WTW and ATA do not correlate, while other studies [16, 20] showed correlations between WTW and ATA. This difference may be caused by different measurement equipment and differences in study subjects; therefore caution is needed to compare studies. These previous studies did not classify by refractive error, and although the correlation trends for each refractive error are similar, our study shows that the correlation may be different. As the WTW was larger, it did not accurately represent the actual angle locations such as ATA and STS [13, 20]. Taechameekietichai et al. used SS-OCT to measure the ATA and STS, and LenStar LS 900 optical biometer to measure the WTW. They showed a significant correlation between WTW, ATA, and STS, and reported that the larger the WTW, the greater the difference between the WTW and the STS [21]. However, in this previous study, only comparative analysis of WTW, ATA, and STS, which correspond to the horizontal meridian, was performed, and the analysis was not performed separately by refractive error. As shown in Table 3, in our study, the correlation coefficients of WTW, ATA, and STS in the horizontal meridian (0, 30, and 150 axes) were larger than in the vertical axis (60, 90, and 120 axes). In our study, the WTW did not appear in proportion to the refractive error, and it can be seen that the difference between WTW, ATA, and STS varies according to the refractive error in Fig 2. In an epidemiological study with a Korean population, WTW was horizontally oval. Horizontal WTW was 11.47 ± 0.55mm and vertical WTW was 10.80 ± 0.60mm in men, while horizontal WTW was 11.17 ± 0.55mm and vertical WTW was 10.48 ± 0.54mm in women [22]. In a study using the postmortem eye, the mean horizontal corneal diameter was 11.46 mm, approximately 0.8 mm greater than the mean vertical corneal diameter of 10.63 mm [23]. Therefore, it is difficult to predict the ATA and STS at all meridians with a horizontally oval WTW.

Although there is a difference in the distribution of ATA and STS between the three refractive groups (Fig 2), it can be seen that the correlation with ATA, STS is not large by analyzing only the refractive error (MRSE) as variable (Fig 3). Consequently, we analyzed factors related to ATA and STS for each meridian by refractive error, as shown in Tables 4 and 5. Edawaji et al. analyzed anterior chamber measurements using OCT, and showed that the STS and anterior chamber angle measurements both increased significantly with increasing age, but the relationship was nonlinear and reached maximum levels by 4 years of age [24]. Iyamu et al. showed the effect of age on corneal diameter (horizontal and vertical WTW) was significant, and the oldest age group had significantly smaller corneal diameter than the younger age groups [25]. Xie et al. used ANTERION SS-OCT with 63 healthy volunteers between 26–74 years of age (spherical equivalent refraction: -8.25 ~ +3.5 diopter), and reported that neither mean ATA distance (R^2^ = 0.06, p = 0.14) or refractive status (R^2^ = 0.04, p = 0.45), nor mean STS (R^2^ = 0.03, p = 0.40) or refractive status (R^2^ = 0.03, p = 0.46) correlated with age [26]. Henriques et al. reported a significant positive association between corneal diameter and AL [27]. In a previous study, ACD and ATA showed a positive correlation in a study of Caucasians and Chinese [28]. Iribarren et al. reported corneal power was negatively correlated with anterior chamber diameter (r = -0.646, P < 0.001), while AL was positively correlated with anterior chamber diameter (r = 0.489, p<0.001) [29]. In a previous study with 43 eyes, using ACD, mean keratometry, WTW, pachymetry, spherical equivalent (mean value: -8.21 ± 9.98 D), and AL as variables to predict sulcus length with UBM, the spherical equivalent and corneal curvature were found to be significant explanatory variables explaining the sulcus length [30]. Corneal curvature is reported to be negatively correlated with sulcus and limbus size (WTW). Compared with our study, there is a difference in the refractive error of the study subject, and the sulcus size measurement method is a point measured using a caliper by combining images captured by UBM rather than measuring STS and ATA. There is a difference in the significant variables that explain ATA and STS. In the case of ATA, unlike STS, ACD has a larger explanatory power than other determinants, and the closer it is to the horizontal axis, the better its explanatory power. In our study, ATA showed a greater positive correlation than STS with measurement parameters indicating the size of the axial axis in the eye, such as AL and ACD. The ACD also showed a statistically significant positive correlation with ATA and STS in most cases; however, the AL showed a statistically significant positive correlation only in the hyperopia group, unlike ATA.

The anterior chamber is thought to be vertically elliptical because the sclera lengthens and thins as the AL becomes longer. There are several studies on the topographic difference of the anterior sclera thickness according to the axial elongation that have shown various correlations, and the distribution of sclera thickness has also varied in previous studies [31–34]. In some previous studies, it was found that the superior [35] and inferior [34] sclera were significantly thinner as the degree of myopia increased, which means that the upper and lower sclera mainly stretched when the AL was elongated. For each meridian, there is a limitation in that the length is not measured between the actual scleral spurs and angle edges. However, in another previous study [36], there was no difference in the volume of sclera, but its thickness decreased as the sheeting field increased. Therefore, as the thickness becomes relatively thin, the length may possibly increase. When the eyes are open, the nasal and temporal sclera receive a cross linking effect by exposure to ultraviolet rays, and the relative rigidity increases [37]. Similarly, the superior and inferior sclera covered with the eyelids do not receive the effect. The increase in eyeball size associated with myopia progression is also related to the change in sclera thickness, and the asymmetry of the thickness change is also related to the anatomy around the eyeball. Atchison et al. showed that the space between the eyeball and the orbital wall was greater in the vertical meridian compared with that of the horizontal meridian based on the findings from MRIs of emmetropic and myopic eyes [38]. Therefore, as myopia progresses, the superior and inferior sclera are elongated more than the nasal and temporal ones, and the angle and sulcus there also elongate backwards, making the anterior chamber vertically oval. As the tendency appears in ATA rather than STS, it can be seen that the corner of the angle becomes longer than the scleral spur as the AL increases.

Considering the previous studies [14–23] on ATA and STS and our study, it seems that there is a clear limitation in predicting the overall structure of the anterior segment with only the horizontal WTW, because there is a difference by location. Because the WTW is horizontally oval and the anterior chamber is vertically oval, the difference between the WTW and anterior chamber in horizontal and vertical meridian can be large, especially in myopia. Thus, the anterior structure cannot be predicted in the same way with different refractive errors with only WTW measured at the horizontal meridian. A previous study showed strong positive correlations between anterior chamber diameter and sulcus diameter that were observed with 35 MHz UBM [12]. However, because the WTW is horizontally oval, and the anterior chamber is vertically oval, conventional estimation of the size of the anterior chamber [39] and ciliary sulcus [40] using horizontal WTW is inadequate. Caution is necessary in judging the size of the ATA, STS, or sulcus to sulcus diameter using only horizontal WTW. In addition, it should be considered that each meridian may have a different correlation depending on the AL or ACD as well as the refractive error.

The results of this study have the advantage of being helpful in predicting the size of the anterior chamber using SS-OCT. In addition, reportedly, there are differences in the anterior segment measurements according with racial differences [11, 41]. Thus, this study is meaningful in that it provides a reference value to know the distribution of ATA and STS by refractive error in Koreans.

There are several possible limitations to the present study. First, it had a retrospective design. Thus, there might have been a selection bias in analysis of each refractive error groups. As a study with Koreans, the range of refraction error in the each study group was relatively wide for myopia group, while the range of refractive error was relatively narrow for hyperpia group. The study subjects are also distributed from the hyperopia group to the elderly, and from the myopia group to the young age. There was a difference in the distribution of ATA and STS between each refractive error group (Fig 2), but the refraction error (MRSE) had little correlation to explain the changes in ATA and STS in total 126 eyes (Fig 3). Previous studies [24–26] also showed various correlations between age and anterior length. In the stepwise multivariate regression analysis of this study, age and refractive error did not show significant explanatory power for ATA and STS. Second, reproducibility and reliability were not evaluated in the measurement of SS-OCT. However, the reproducibility and reliability of ANTERION SS-OCT has already been established in previous studies [5, 26, 42–44]. Additionally, we believe that the error that may occur was minimized due to the fact that all three acquisition quality parameters (“Motion,” “Fixation,” and “Tear film and lid”) were required to receive a "pass" result. Third, all the subjects in the study were Korean. Our results may not be generalizable to other ethnicities. Since there are differences in the size of the eyeball, WTW difference, and refractive error distribution among races, it is likely that it will be difficult to generalize and apply the results of this study to other races. However, presenting reference values of ATA and STS and analysis by refractive error in various meridian axes in Koreans has its own advantages. Fourth, the diameter of the sulcus-to-sulcus was not measured directly. Currently, although a light source with the deep penetration of SS-OCT is used, equipment that shows the entire sulcus diameter is not commercially available. However, it is thought that the measurement of ATA and STS for each meridian is more helpful in indirectly predicting the sulcus-to-sulcus diameter than WTW. Lastly, only the horizontal WTW was compared with the ATA and STS of other meridians. In general, WTW is measured automatically on the horizontal axis by many biometries, and it is known from previous studies that it has a horizontally oval WTW distribution. In the future, it will be necessary to measure the WTW for each meridian and to study the correlation with the ATA and STS of each meridian.

In conclusion, this study is the first to analyze the relationship between ATA and STS compared to horizontal WTW by dividing them into groups of myopia, emmetropia, and hyperopia by multiple meridians and to show the patterns. The angle diameter, expressed as ATA and STS, is a vertically oval shape but differs from horizontal WTW for each refractive error. It should be known that each refractive error group has a different correlation in the prediction of the angle diameter and the sulcus-to-sulcus diameter using only horizontal WTW. It is thought better to estimate ATA and STS by considering variables such as ACD or AL associated with eyeball size. Additionally, this study is meaningful in that it provides reference values of ATA and STS by refractive error in Koreans. Further analysis with the sulcus-to-sulcus size is needed with a larger study group in the future.

## Supporting information

S1 File Data analyzed. (XLSX)
